# Thermodynamic stability, in-vitro permeability, and in-silico molecular modeling of the optimal *Elaeis guineensis* leaves extract water-in-oil nanoemulsion

**DOI:** 10.1038/s41598-021-00409-0

**Published:** 2021-10-21

**Authors:** Nissha Bharrathi Romes, Roswanira Abdul Wahab, Mariani Abdul Hamid, Habeebat Adekilekun Oyewusi, Nurul Huda, Rovina Kobun

**Affiliations:** 1 Department of Chemistry, Faculty of Science, Universiti Teknologi Malaysia, 81310 UTM Johor Bahru, Malaysia; 2 Enzyme Technology and Green Synthesis Group, Faculty of Science, Universiti Teknologi Malaysia, 81310 UTM Johor Bahru, Malaysia; 3 School of Chemical and Energy Engineering, Faculty of Engineering, Universiti Teknologi Malaysia, 81310 UTM Johor Bahru, Malaysia; 4 Faculty of Food Science and Nutrition, Universiti Malaysia Sabah, Kota Kinabalu, 88400 Sabah, Malaysia

**Keywords:** Biotechnology, Computational biology and bioinformatics, Molecular biology, Chemistry, Engineering, Nanoscience and technology

## Abstract

Nanoemulsion is a delivery system used to enhance bioavailability of plant-based compounds across the stratum corneum. *Elaeis guineensis* leaves are rich source of polyphenolic antioxidants, viz. gallic acid and catechin. The optimal *E. guineensis* leaves extract water-in-oil nanoemulsion was stable against coalescence, but it was under significant influence of Ostwald ripening over 90 days at 25 °C. The in-vitro permeability revealed a controlled and sustained release of the total phenolic compounds (TPC) of EgLE with a cumulative amount of 1935.0 ± 45.7 µgcm^−2^ after 8 h. The steady-state flux and permeation coefficient values were 241.9 ± 5.7 µgcm^−2^ h^−1^ and 1.15 ± 0.03 cm.h^−1^, respectively. The kinetic release mechanism for TPC of EgLE was best described by the Korsmeyer–Peppas model due to the highest linearity of R^2^ = 0.9961, indicating super case II transport mechanism. The in-silico molecular modelling predicted that the aquaporin-3 protein in the stratum corneum bonded preferably to catechin over gallic acid through hydrogen bonds due to the lowest binding energies of − 57.514 kcal/mol and − 8.553 kcal/mol, respectively. Thus, the in-silico study further verified that catechin could improve skin hydration. Therefore, the optimal nanoemulsion could be used topically as moisturizer to enhance skin hydration based on the in-silico prediction.

## Introduction

Nanoemulsion is an emulsion typically made up of oil, surfactant, and water with nanometer-sized droplets. To be more specific, the droplet size varies depending on the authors, with some contemplating 500 nm as the upper limit. Depending on the droplet size, a nanoemulsion can be categorized into two groups, (i) the transparent or translucent (< 300 nm) and milky (up to 500 nm)^1,2^. There are oil-in-water or water-in-oil nanoemulsions, whereby the former consists of oil droplets dispersed within a water phase. Whereas the latter consists of water droplets dispersed in an oil phase^3^. Notably, nanoemulsions are kinetically stable but thermodynamically unstable, which means that the systems undergo destabilization phenomena such as creaming, sedimentation, flocculation, coalescence, and Ostwald ripening, eventually leading up to phase separation^4^. Nanoemulsions are excellent and promising topical delivery systems (TDS) to improve the bioavailability of poorly soluble plant-based bioactive compounds to targeted areas. The minute droplets in the nanoemulsion system encapsulate the plant-based bioactive compounds with low solubility in water or oil phases. The system also simultaneously protects the compounds from physical and chemical degradation, enable their controlled and sustained release at the target site^5^.

However, the epidermis's stratum corneum (SC) is a barrier to the passage of bioactive molecules. It reduces the permeation efficiency of bioactive compounds through the SC^6^. Due to this, nanoemulsions intended for TDS should focus on in-vitro permeability efficacy. The technique could provide reliable efficacy or bioavailability data on the formulated nanoemulsion in TDS. Therefore, skin permeation of nanoemulsion is an important aspect for TDS to ensure the encapsulated bioactive compounds are released successfully across SC to bind or react accordingly to treat the illness or repair the damaged cells^7^. The permeation of nanoemulsions through the skin is closely related to the AQP-3 protein. It is a water channel-expressed enzyme predominantly found in the epidermis layer of human skin and plays a key role in skin moisturization. The protein is highly expressed in keratinocytes of the stratum corneum, which is fundamental for skin hydration. In fact, the AQP-3 protein is a protease that facilitates the transportation and proliferation of keratinocytes in the epidermis for wound healing. Literature has shown that the AQP-3 protein expression level decreased with aging, causing substantial skin dryness^8^.

### *Elaeis guineensis*

Jacq. is a perennial plant grown abundantly in tropical countries, such as Malaysia and Indonesia. The biomass from the oil palm industry ranges from oil palm leaves, oil palm fronds, oil palm trunks, empty fruit bunches, palm kernel shells, mesocarp fibers and palm oil mill effluent which are poorly explored or under-utilized^9,10^. In particular, *E*. *guineensis* leaves extract (EgLE) are a rich source of various hydrophilic polyphenols, viz. gallic acid, catechin, and their derivatives. In our previous study, these water-soluble antioxidants, gallic acid, and catechin were encapsulated and formulated into an optimal EgLE water-in-oil nanoemulsion, and the physicochemical properties were characterized. The atomic force micrograph confirmed the droplet sizes as 180.0 nm, 146.6 nm and 148.6 nm^11^. Due to the poor lipid solubility of these hydrophilic polyphenols, the study chose the water-in-oil type of nanoemulsion to encapsulate them for better penetration and delivery by transepidermal absorption. That was the first ever attempt on the optimal nanoformulation loaded with EgLE as the bioactive ingredients for topical application to the best of our knowledge. Subsequently, the thermodynamic stability, in-vitro permeability as well as in-silico molecular modeling of the optimal nanoemulsion are reported and discussed in detail in this study.

## Material and methods

### Plant material

Fresh *E. guineensis* leaves were collected throughout October 2017 from an oil palm plantation on the grounds of Universiti Teknologi Malaysia, Johor with the permission from the research’s Supervisor. The plant experiment was carried out in accordance with the guidelines. A voucher specimen of H061 has been deposited at the Herbarium of Universiti Putra Malaysia in 2021. The specimen was identified and confirmed by Mrs. Rosni Ludin. The collected samples were air–dried for a week, cut, and ground into smaller pieces. The samples were sealed in the ziplocked plastic bags and stored at ambient temperature (25 °C) until further use.

### Chemicals and reagents

Folin–Ciocalteu's phenol reagent was purchased from Sigma–Aldrich (St. Louis, MO, USA). Strat-M membrane, disodium hydrogen phosphate, potassium dihydrogen phosphate, sodium chloride, potassium chloride, and sodium carbonate were purchased from Merck (Darmstadt, Germany). The olive oil and sunflower seed oil were purchased in Johor Bharu, Malaysia. (Hydroxypropyl)methylcellulose and Brij L23 (polyoxyethylene (23) lauryl ether) (HLB = 16.9) were bought from Sigma–Aldrich (St. Louis, MO, USA), whereas Span 80 (sorbitan monooleate) (HLB = 4.3) was purchased Sisco Research Laboratories (Maharashtra, India). 2-Phenoxyethanol (antimicrobial agent) and vanilla perfume were procured from Personal Formula Resources, Johor Bahru, Malaysia. Analytical grade ethanol of 99.86% mass fraction purity was bought from Haymankimia (England). Purified water was prepared using an 18 mΩ Millipore Milli–Q deionized water purification system. UV–visible spectrophotometer (UV-1601PC, Shimadzu) and analytical balance Shimadzu Philippines Manufacturing Inc. (Philippines) was used for measuring wavelength and weighing, respectively.

### Preparation of *E. guineensis* leaves extract

The dried and finely ground leaves (1 g) were weighed and transferred into centrifuge tubes (50 mL) containing 25 mL of 50% v/v ethanol. The suspensions were homogenized using the homogenizer IKA T18 Digital Ultra Turrax (Germany) at 10,000 rpm for 40 s. The samples were then subjected to ultrasonic-assisted extraction using an ultrasonicator probe Sonics Vibra Cell (America) performed for 30 min, at 20 kHz frequency, constant power of 130 W, at sonication amplitude of 60% and constant temperature of 25 °C. Sample collection was done by centrifuging each sample mixture for 15 min at 4,000 rpm, and the supernatants were collected and filtered through a Whatman No. 1 filter paper. The solvent was removed using a rotary evaporator under reduced pressure at 40 °C to obtain the crude extract and then lyophilized for 48 h. Finally, the crude extracts were weighed and kept at 4 °C until further analysis.

### Preparation of the optimal nanoemulsion

The optimal nanoemulsion was prepared by the high-energy method, ultrasonication. The oil phase was a mixture of olive oil and sunflower seed oil in the ratio of 1:4. A 1:4.7 ratio blend of two non-ionic surfactants mixture, Brij L23 and Span 80, was prepared to a final HLB value of 6.5. The oil phase (10% w/w) containing the surfactant mixture (29% w/w) was heated up to 80 °C with stirring at 700 rpm (0.02%, w/w) of EgLE was dissolved in the aqueous phase by ultrasonication in an ultrasonic bath (110 W and 40 kHz, Branson 3800, Mexico) for 30 min. 0.6% (w/w) of hydroxylpropylmethylcellulose was added to the aqueous phase while stirring continuously in an ice bath for 5 min at 700 pm. The oil phase was then added dropwise to the aqueous phase (58% w/w) while stirring continuously at 15,000 rpm using the IKA T18 Digital Ultra Turrax (Germany), followed by the addition of 0.7% (w/w) of 2-phenoxyethanol and 1.68% (w/w) of vanilla perfume. Lastly, the coarse nanoemulsion was ultrasonicated for 1 h at 60% sonication amplitude using an ultrasonic probe Sonics Vibra Cell (USA), to prepare the optimally formulated nanoemulsion.

### Monitoring of MDS, PDI, and zeta potential for thermodynamic stability

The optimal nanoemulsions used in this work were prepared by osbservaing their physicochemical characteristics in our previous study^11^. Consequently, the optimal nanoemulsion was assessed monthly for thermodynamic stability in terms of MDS, PDI, and zeta potential for 90 days storage period at 25 °C in this study. These parameters were measured simultaneously using Zetasizer Nano ZSP (Malvern Instruments, United Kingdom). Each sample was dissolved in deionized water and transferred into a cuvette capillary cell for analysis. The most important parameter in this study, the MDS data, was collected for over 3 months. The information is useful to estimate destabilization phenomena related to coalescence and Ostwald ripening in the nanoemulsion, when stored for an extended duration.

### Rate of coalescence

The rate of coalescence of the optimal nanoemulsion was determined in terms of MDS over 90 days of storage stability study at 25 °C using Eq. (1):

1 $$\frac{1}{{r^{2} }} = \frac{1}{{r_{o}^{2} }} - \left( {\frac{8\pi }{3}} \right)\omega t$$

For Eq. (1), *r* refers to the mean radius after time, *r*_*o*_ represents the value at a time (s) t = 0. Meanwhile, ω refers to rupture frequency per unit of the film surface. This study plotted the graph of 1/*r*^2^ versus storage time (s) to observe the rate of coalescence. A linear relationship graph was predicted for the optimal nanoemulsion influenced by the coalescence rate.

### Rate of ostwald ripening

The Lifshitz–Slesov–Wagner theory was used to determine the Ostwald ripening rate for the optimal nanoemulsion using Eq. (2). Ostwald ripening is instigated when the system's droplet size increases after a certain duration due to the oil phase diffusing through the aqueous phase.

2 $$\omega = \frac{{dr^{3} }}{dt} = \frac{8}{9}\left[ {\frac{{C(\infty )V_{m} D}}{\rho RT}} \right]$$

where ω denotes the frequency of rupture per unit surface of the film, *r* represents the average radius of droplets over time, *t* refers to the storage time (s), and *C* (∞) is the bulk phase solubility. The term *V*_*m*_ is the molar volume of the internal phase, *D* denotes the diffusion coefficient of the dispersed phase in the continuous phase, ρ is the dispersed phase's density, *R* refers to the gas constant, and *T* is the absolute temperature. Ostwald's ripening rate of the optimal nanoemulsion was observed by plotted the *r*^3^ versus storage time (s).

### Heavy metal analysis

Heavy metal analysis was done on the optimal nanoemulsion to determine the presence of arsenic, cadmium, and lead. The analysis used a Mercury Analyzer according to AOAC 968.08 (Sample Preparation) & USEPA 6010B. Meanwhile, the mercury quantification test was done according to the in-house method LWI-MFF 027 based on USEPA 7473.

### Antimicrobial assay

Antimicrobial assays for the optimal nanoemulsion to confirm the presence of *Candida albicans*, *Pseudomonas aeruginosa*, S*taphylococcus aureus*, Total Yeast & Mould, and Total Plate Count were performed following the FDA-BAM^12^.

### In-vitro permeability

The diffusion of the optimal nanoemulsion across the mimic skin-like membrane, Strat-M membranes (Millipore, Temecula, MA, USA) was investigated using Franz diffusion cells (Perme Gear, Hellertown, PA, USA), which consist of two main compartments, receptor, and donor. The membrane (25 mm, 300 μm) was placed between both compartments, and a clip was used to avoid leakage of the nanoemulsion sample. The previously filled receptor compartment with phosphate buffer saline (PBS) (pH 7.4):ethanol (1:1) (7 ml) was stirred continuously with a magnetic stirrer at 37 °C. The diffusional area of the receptor compartment was 1.77 cm^2^. The optimal nanoemulsion (3 ml) sample was loaded onto the donor compartment homogeneously. A 0.5 ml aliquot of the sample was withdrawn from the receptor compartment at a fixed time intervals (0, 1, 2, 3, 4, 5, 6, 7, and 8 h) and replaced with the same volume of PBS:ethanol solution. The samples were assayed for total phenolic compounds (TPC) using Folin-Ciocalteu (FC) reagent by UV–Visible spectrophotometer (UV-1601PC, Shimadzu) with slight modifications. Approximately 0.2 mL aliquots of the hourly sample was mixed with the FC reagent (0.2 mL) and deionized water (1.8 mL). After 5 min, 7% (w/v) Na_2_CO_3_ (2 mL) and deionized water (0.8 mL) are added to the mixture. The extracts were then incubated for 30 min before the absorbances read at 765 nm ^9^. All experiments were triplicated in this study. The calibration curve with the concentrations of 0–400 µg/ml for the TPC of the EgLE was constructed. The cumulative percentage of the TPC of EgLE permeated through the synthetic membrane per unit area was plotted as a function of time. The permeation rate at a steady-state (Jss, μgcm^−2^ h^−1^) was calculated from the slope of the linear function of the cumulative amount of the TPC of EgLE permeated per unit area versus time. The permeability coefficient, Kp, was determined using Eq. (3):

3 $${\text{Kp}} = {\text{J}}/{\text{Co}}$$

where J is the skin permeation rate, and Co is the concentration at the donor compartment.

### Kinetic release study

The kinetic release mechanism of the TPC of EgLE from the optimal nanoemulsion was determined based on the known five mathematical models typically used to describe the release behavior of polyphenols (gallic acid and catechin) from the nanoemulsions. The models were the zeroth-order (cumulative percentage of drug release against time, Eq. (4)), first-order (log cumulative percentage of drug remaining against time, Eq. (5)), Higuchi (cumulative percentage of drug release against the square root of time, Eq. (6)), Hixson-Crowell (cube root cumulative percentage of drug remaining against time, Eq. (7)) and Korsmeyer-Peppas (log cumulative percentage of drug release against log time, Eq. (8))^13,14^. The best fitted kinetic release model for the optimal nanoemulsion was chosen based on the highest coefficient determination, R^2^ obtained from all the plotted graphs.

In the following Eqs. (4)–(8), *M*_t_ is the amount of the TPC of EgLE released over time *t*, *M*_0_ is the initial amount of the TPC of EgLE in the dissolution media, *M*_left_ is the amount of the TPC of EgLE left in the solution over time, *M*∞ is the amount of the TPC of EgLE released after an infinitive time (in this study after 8 h), and *n* is the parameter indicative of the release mechanism. Meanwhile, *k*_0_, *k*_1_, *k*_H_, *k*_w_, and *k*_K-P_ are the zero-order release, first-order release, Higuchi, Hixson-Crowell, and Korsmeyer-Peppas rate constant, respectively.

4 $$M_{{\text{t}}} = M_{0} + k_{0} {\text{t}}$$

5 $${\text{ln }}\left( {M_{0} - M_{{\text{t}}} } \right) \, = {\text{ ln}}M_{0} + k_{{1}} t$$

6 $$M_{{\text{t}}} = k_{{\text{H}}} \sqrt t$$

7 $$\sqrt[3]{{M_{0} }} = \sqrt[3]{{M_{left} }} + k_{w} t$$

8 $${\text{log }}\left( {M_{{\text{t}}} /M\infty } \right) \, = {\text{ log}}k_{{{\text{K}} - {\text{P}}}} + n{\text{log}}t$$

### Characterization of the AQP-3 protein

The FASTA sequence of the amino acid of AQP-3 protein (GenBank accession number: AAY68214.1) was downloaded from the National Center for Biotechnology Information (NCBI) database (https://www.ncbi.nlm.nih.gov/). Next, the sequences with the highest sequence similarity in the Protein Data Bank (PDB) were identified with NCBI's BLAST. The retrieved amino acid sequence of the AQP-3 was modeled using the online SWISS-MODEL software (https://swissmodel.expasy.org/) to generate its 3D structure. The generated AQP-3 model was saved in PDB format and viewed by Pymol.

The molecular structure of the AQP-3 was energy minimized to get a correct stereochemistry and achieve a molecular structure to resemble closely with the native structure. This was done on the GROMACS 2018.6 using the steepest descent and conjugate gradient methods (https://www.gromcs.org/About_Gromacs). The 3D structure of the AQP-3 protein was evaluated before and after energy minimization to assess the quality of the constructed model using several tools provided in the UCLA-DOE server (http://servicesn.mbi.ucla.edu/). The protein model was validated by PROCHECK for stereochemical quality and illustrated in a Ramachandran plot. In contrast, Verify 3D was used to determine the compatibility of an atomic model to its own amino acid. Lastly, the protein structures from non-bonded interaction were verified by ERRAT^15,16^.

### Characterization of the ligands

The substrates 3D structures for the subsequent molecular docking and molecular dynamic simulations used ligands, gallic acid, and catechin retrieved from the PubChem database (3D modeling conformer). The SDF files of the ligands were converted to PDB files (version 2.3.1) by OPENBABEL before using the Avogadro software to add hydrogens to the 3D model of the ligands. Finally, the structures were submitted to the Automated Topology Builder (ATB) and Repository server version 3.0 to optimize the geometries of the molecules^17^.

### Molecular docking

Molecular docking was done using the Autodock version 4.2.6 to predict the possible docking energies, sites, and binding interaction. Water molecules were first excluded from the AQP-3 protein, followed by polar hydrogens and nonpolar hydrogens before the total Kollman and Gasteigher charges were assigned. Here, the best result for each substrate was chosen as the largest conformation cluster registering the lowest binding energy (kcal/mol). It is reported earlier that the lowest binding energy represents a simple estimation of a better or even stronger ligand affinity towards the protein for protein–ligand complex formation^18^. Finally, the "pdbqt" file for each AQP-3 ligand complex was converted to the "pdb" format and visualized by PyMOL (version 2.4.1 Schrodinger, LLC) to identify the amino acid positions alongside their hydrogen bond distances. The LigPlots exhibit both hydrogen bonds and hydrophobic interactions of each AQP-3 ligand complex.

### Molecular dynamic simulation

The molecular dynamic (MD) simulation analysis of the protein–ligand is used consecutively with molecular docking to confirm the stability of the docked complexes. The MD simulation was examined based on Root Mean Square Deviation (RMSD), Root Mean Square Fluctuation (RMSF), and Radius of gyration (Rg) values as a function of time. RMSD and RMSF can generally measure the dynamic behavior and structural changes of the AQP-3 protein^19^. RMSD denotes conformational changes and stability of the protein–ligand complex. Hence, a low RMSD value signifies a stable AQP-3-ligand complex and vice versa.

Conversely, residues contributing to complex structural fluctuations can be assessed by the RMSFs of each residue. The RMSF value denotes the complex flexibility with respect to each amino acid movement. A high RMSF value presents a higher degree of movement or flexibility, whereas a low RMSF value indicates the high stability of the structure due to limited movement during simulation. Rg value describes a stable folded structure and is related to the compactness changes of a ligand–protein complex. Most importantly, the Rg value offers an insight into the overall dimension and the shape of the protein–ligand complex. Hence, a relatively similar Rg value between complexes implies the protein's general shape was stable or unaltered upon binding the ligand^17^.

As for the validation of MD simulation results, calculating the free binding energy of the AQP-3 protein with the ligands was carried out using the g_mmpbsa tool in conjunction with the GROMACS package coupled with Adaptive Poisson-Boltzmann Solver (APBS). A g_mmpbsa package is a standalone tool containing the GROMACS and APBS package subroutines that provide a detailed estimation of the Poisson-Boltzmann surface area's molecular mechanics (MM-PBSA) interaction.

## Results and discussion

### Monitoring of MDS, PDI, and zeta potential for thermodynamic stability

**Figure 1:**
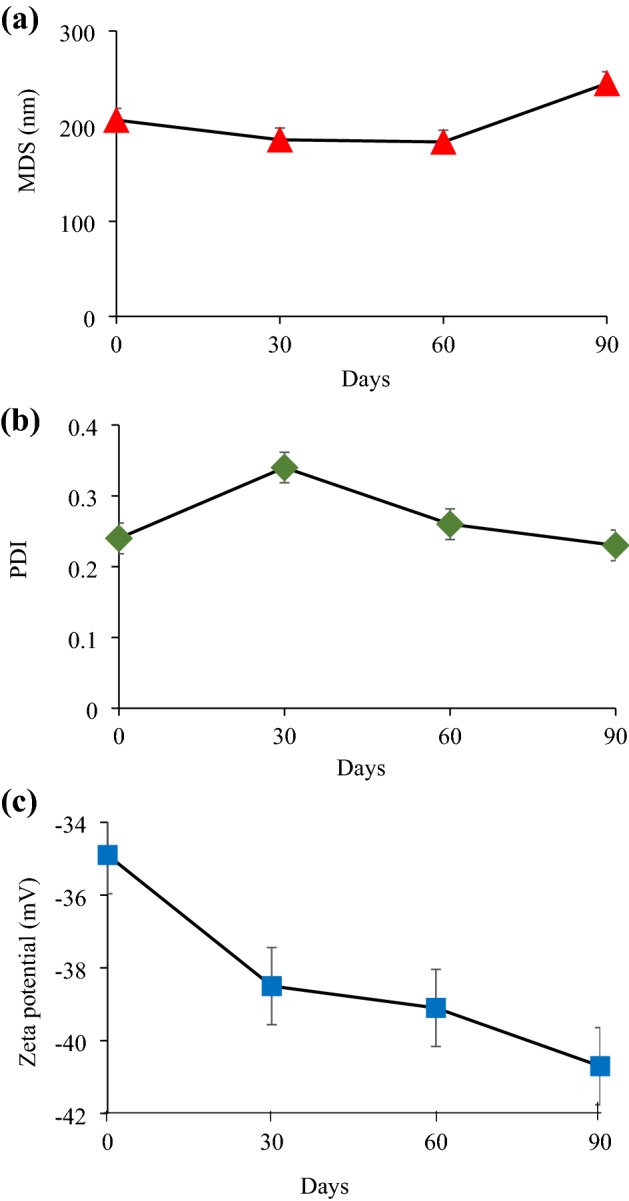
Results of the 90 days storage stability of the optimal nanoemulsion at 25 °C, where plot (**a**) Mean droplet size (MDS), (**b**) Polydispersity index (PDI), and (**c**) zeta potential.

The stability of nanoemulsions is an important character that explains the shelf life of any given formulation. Monitoring of MDS, PDI, and zeta potential during 90 days storage at 25 °C is crucial for the optimal nanoemulsion to determine the thermodynamic stability of the system against coalescence and Ostwald ripening phenomena^20^. Figure 1 illustrates the changes in MDS, PDI, and zeta potential of the optimal nanoemulsion within 90 days storage period at 25 °C. MDS of the optimal nanoemulsion on the first day of preparation was slightly higher than the acceptable range (< 200 nm) (Fig. 1a). This was likely due to the colloidal dispersion system that has yet to stabilize and reach equilibrium. The same trend was observed by another study that prepared a glutathione-loaded water-in-oil nanoemulsion where the MDS increased steadily after 30, 60, and 90 days, showing values of 96.05 nm, 155 nm, 181.3 nm, and 190 nm, respectively.

Furthermore, the stability of the said nanoemulsion was closely associated with the nanodroplets. Ostwald ripening caused the increase in MDS, thus increasing the tendency to undergo phase separation. A drastic rise in MDS indicates low stability of the nanoemulsion system^21^. The MDS of the optimal nanoemulsion was maintained around 180 nm until the 60th day. However, there was an increase in MDS on 90th day to 244.8 nm, likely due to two phenomena: coalescence or Ostwald ripening. This is because the surfactant type and its composition in the formulation play an important for optimal nanoemulsion stabilization. The surfactant is essential to ensure that the water phase is distributed into very small droplets. The outcome can be achieved in the optimal nanoemulsion by the surfactant mixture (Brij L23, Span 80) encompassing and protecting the water droplets from coalescence^22^.

As can be seen in Fig. 1b, PDI values until 90 days of storage stabilized at ~ 0.25, hence well within the acceptable benchmark (< 0.25) for a stable nanoemulsion system^23^. The results thus communicated that the optimal nanoemulsion still exhibited a monodisperse characteristic. The good stability seen in the optimal nanoemulsion was likely contributed by surfactant in the colloidal dispersion system. Contrariwise, the PDI was 0.34 on the 30th day, slightly higher than the acceptable specification. The outcome seen here was presumably due to a certain percentage of the droplets in the nanoemulsion system combining to form slightly bigger droplets. A study was done by Sulaiman et al. observed that the PDI on the 30th day decreased slightly compared to the 1st day. In short, the said nanoemulsion maintained a PDI below 0.25 throughout the 90 days storage period^22,24^.

As can be seen in Fig. 1c, the optimal nanoemulsion's zeta potential changed between − 30.0 to − 40.0 mV over 90 days. This demonstrated the optimal nanoemulsion's adequate stability to overcome destabilization phenomena through the repulsive forces formed between negatively charged nano-sized water droplets. The optimized nanoemulsion results indicated the good physical stability of its dispersed systems, as similarly observed by a similar work^25^. Previously, studies have shown that the zeta potential of any given nanoemulsions ought to be highly negatively charged for good stability. A water-in-oil nanoemulsion described by a recent study showed that droplets with a surface charge would get attracted to the droplets of opposite charge, which then formed an electric double layer. The zeta potential of the nanoemulsion was –32.0 mV to –38.2 mV. This proved that the negatively charged droplets were highly stable to overcome coalescence, flocculation, creaming, Ostwald ripening, or sedimentation^26^. Strong repulsive forces between the nano-sized droplets in the colloidal dispersion system cause a highly negative/positive zeta potential. This agreed with an observation by Roselan et al., which obtained a nanoemulsion loaded with kojic monooleate with a zeta potential of − 30 mV after 90 days of storage^27^.

### Rate of coalescence

Notably, nanoemulsions are proven to be capable of withstanding all types of storage instabilities viz. creaming, sedimentation, aggregation, flocculation, coalescence, and Ostwald ripening compared to microemulsions due to nano-scaled droplets. Nevertheless, their stability could depreciate with time via two well-known distinctive irreversible demulsification processes. They are the Brownian-induced coalescence and Ostwald ripening. Coalescence describes a storage instability phenomenon involving two or more droplets fusing into bigger droplets in nanoemulsion systems^28^. For instance, the optimal nanoemulsion could undergo a coalescence effect over time as the small water droplets tend to collide with each other. Given enough time, this increases the MDS of the system and affecting the storage stabilities, and eventually result in phase separation.

**Figure 2:**
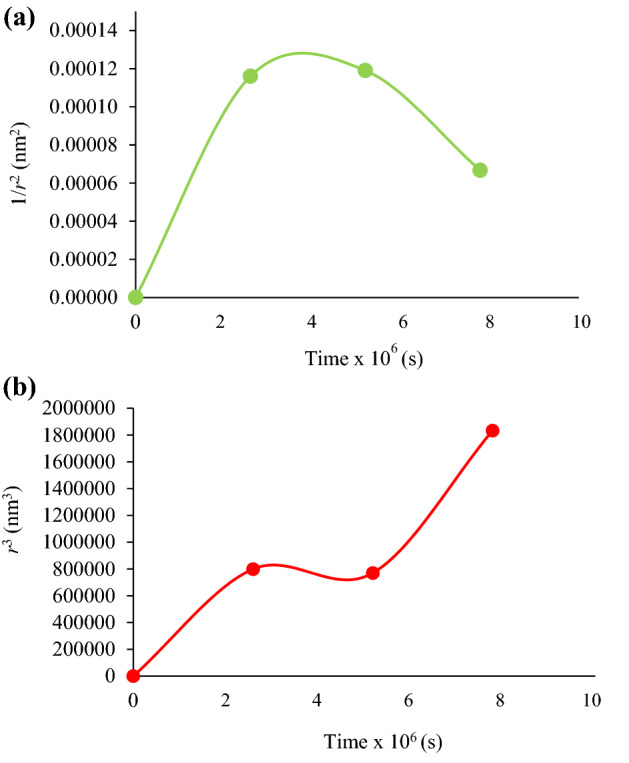
(**a**) Rate of coalescence and (**b**) Rate of Ostwald ripening graphs in terms of MDS (mean droplet size) for the optimal nanoemulsion under 90 days of storage at 25 °C.

In this study, the coalescence rate of the optimal nanoemulsion was estimated by plotting the reciprocal of the *r*^2^ of the MDS obtained from Fig. 1a. A linear graph is obtained for a coalescence affected nanoemulsion stored over time, causing the MDS to increase linearly with increasing time. Figure 2a depicted the plotted graph of 1/*r*^2^ versus storage time was not linear, revealing that the optimal nanoemulsion stored at 25 °C was stable. The overall trend seen here indicated that the change in MDSs as a function of time was not coalescence-related^29^. A plausible explanation might be due to the contribution of the surfactant mixture, Span 80 and Brij L23, used in this optimal nanoemulsion. The results thus indicated that both emulsifiers were adequate in preventing droplets from coalescing due to the high viscosity. The wax-like Brij L23 and the highly lipophilic Span 80 were good emulsifiers for coating the water nanodroplets. These surfactants restrict droplets' movements and avert coalescence^30^.

### Rate of Ostwald ripening

Alternatively, another stability degradation mechanism in nanoemulsion is Ostwald ripening. This process involves the irreversible growth of larger droplets in the nanoemulsions at the expense of smaller ones. In fact, nanoemulsions with a considerably soluble dispersed phase in the continuous phase are prone to destabilization by Ostwald ripening. Ostwald ripening differs from coalescence because the former does not need any contact between the droplets. The changes mentioned above are brought about by the difference in Laplace pressure inside the droplets^31^. Figure 2b presents the plotted graph of *r*^3^ versus storage time, which revealed that the optimal nanoemulsion stored at 25 °C was under significant influence of the Ostwald ripening, based on the increase in MDS from 206.3 nm on day-1 to 244.8 nm on day-90. However, the *r*^3^ value of the optimal nanoemulsion somewhat plateaued between the 30th and 60th day. In the first 2 months, the nanoemulsion stability likely resulted from the lipophilic surfactant Span 80 that halted the Ostwald ripening effect. The surfactant molecules restricted the water droplets' diffusion into the continuous oil phase.

On the other hand, the increasing MDS in the last 30 days of this study was due to the diffusion of the smaller water droplets through the bigger water droplets (Fig. 2b). It was apparent that the ambient temperature promoted the Ostwald ripening phenomenon in the optimal nanoemulsion, related to the water droplets' high kinetic energy in the dispersion phase that increased their movement^32^. The outcome seen above was in good agreement with the earlier report by Gupta et al. They found the oil phase's chemical potential (sunflower seed and olive oils) was higher in smaller droplets than in bigger ones. This factor allowed the mass transfer of the smaller oil droplets to the larger droplets^33^. A curcumin-loaded MCT nanoemulsion prepared by Kim et al. also demonstrated similar behavior. In their work, the sorbitan monooleate/Span 80 (HLB: 4.3) was the selected emulsifier, which successfully retarded the Ostwald ripening phenomenon toward the MCT oil phase by forming an interfacial layer, possibly from within the droplets^34^.

### Heavy metal analysis

Nanocosmeceutical products should be free from the presence of heavy metals such as arsenic, cadmium, lead, and mercury due to their high toxicity, skin irritation, and cancerous effect on consumers. The FDA has set acceptable limits for these heavy metal presence in cosmetics which are arsenic (< 3 ppm), cadmium (< 0.3 ppm), lead (< 20 ppm), and mercury (< 1 ppm) (FDA, 2018)^35,36^. In this study, the heavy metal analysis on the optimal nanoemulsion confirmed that arsenic, cadmium, lead, and mercury was detected at low levels, namely < 0.5 ppm, < 0.1 ppm, < 0.5 ppm < 0.1 ppm, respectively. The results indicated that the ultrasonically extracted polyphenolic compounds (gallic acid and catechin) from the *E. guineensis* leaves in 50% ethanol:water yielded safe active ingredients for the topically applied optimal nanoemulsion. The heavy metal analysis results corroborated that the leaves extract was safely purified, thus indicating the extraction procedure's suitability to isolate the polyphenolic compounds from the raw oil palm leaves.

It is pertinent to indicate here that there are frequent reports about the heavy metals in creams, mostly from the contaminated raw material. This was due to the lack of compliance by small-scale manufacturers and the lack of strict FDA regulations. Specifically, color additives and perfumes may contain these residual impurities (arsenic, cadmium, lead, and mercury), which eventually contaminate the nanoemulsions during preparation^37^. Henceforth, the aforementioned heavy metals are carcinogenic and prohibited in cosmetics. In conclusion, the optimal nanoemulsion is safe for topical use because the heavy metals are within the allowable range^35^.

### Antimicrobial assay

Topical nanoemulsion should be investigated for antimicrobial assay to determine their inhibitory effects against different microorganisms or gauge whether the emulsions formulation might promote microbial growth. The cosmetic products are usually stored at varying storage conditions, largely between 4 °C to 25 °C. Cold and ambient environments are suitable for microbial growth in any medium; thus, it is important to test the cosmetics^38^. The antimicrobial assays confirmed the absence of *Candida albicans*, *Pseudomonas aeruginosa*, *Staphylococcus aureus*, Total Yeast & Mould, and Total Plate Count in the optimal nanoemulsion. These microorganisms were used for the study's antimicrobial assay as they are the microbial agents of concern in cosmetic products, which might degrade and jeopardize the product's quality and safety.

The Total Plate Count and Total Yeast & Mould colonies were detected below 10 CFU/g in the optimal nanoemulsion, while growths of *S. aureus*, *P. aeruginosa,* and *C. albicans* were not detected on the test plates. Henceforth, the outcome corroborated that the optimal nanoemulsion is an unsuitable medium for pathogen growth if kept in cold and/or ambient environments during long-term storage. As a precaution, the added antimicrobial agent, 2-phenoxyethanol, probably inhibited pathogen growth in the optimal nanoemulsion.

Supposedly, the nanoemulsions were not assayed for antimicrobial properties. However, pathogenic Gram-positive bacteria and Gram-negative bacteria might thrive in them during prolonged storage conditions. This is because warm temperatures are suitable environments for the growth of pathogenic bacteria^39,40^. Also, the larger surface area in smaller droplets promotes their greater interaction with microbial cells^41^.

### In-vitro permeability

**Figure 3:**
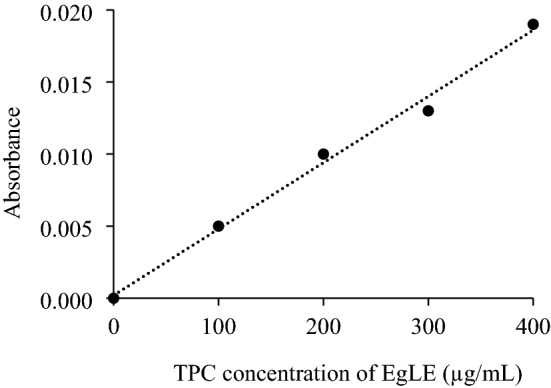
Calibration curve for TPC of EgLE.

**Figure 4:**
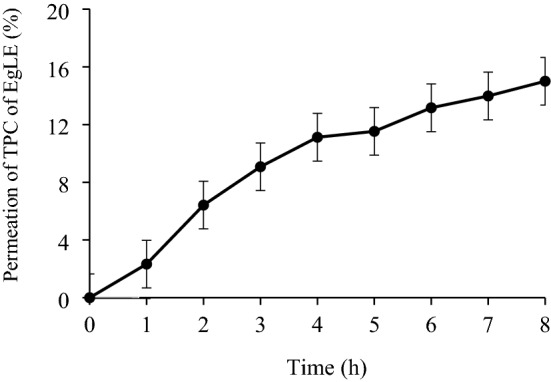
Percentage of the TPC of EgLE permeated from the optimal nanoemulsion after 8 h.

The in-vitro permeability of the optimal nanoemulsion was carried out to study the bioavailable amount of polyphenolic compounds for skin penetration when applied topically^42^. The commercially available synthetic Strat-M membrane was used in this in-vitro study. This is due to its multiple polyethersulfone layers that are morphologically similar to the human skin and have a very tight surface layer. This synthetic membrane is composed of two layers of polyethersulfone lying on top of another layer of polyolefin. This membrane type is the most suitable synthetic membrane to simulate human skin for active compounds or drug diffusion experiments^43^. The calibration curve of the TPC of EgLE (R^2^ = 0.9925) (Fig. 3) was constructed to determine the amount of the TPC of EgLE diffused through the Strat-M membrane from the donor compartment to the receptor compartment of the Franz diffusion cell. The amount of TPC of EgLE permeated hourly was determined by multiplying the volume of the receptor compartment with the concentration of the corresponding hourly sample obtained from the plotted calibration curve, followed by division of the yielded amount with the diffusional area. As can be seen, the TPC of EgLE permeation increased with increasing time, reaching up to 15% of the loaded nanoemulsion after 8 h (Fig. 4). Overall, the TPC of EgLE permeated across the membrane almost in a linear relationship, indicating a controlled and sustained release of the TPC of EgLE from the optimal nanoemulsion.

Literature has shown that the active ingredients' properties and the delivery system's type are factors that influence their controlled release from the nanoemulsion^13^. As seen in this study, the permeation of the targeted compounds of EgLE, namely, gallic acid and catechin, in the form of encapsulated water droplets from the optimal nanoemulsion through the Strat-M membrane increased as a function of time. The outcome seen here was likely due to their minute droplet size and low PDI alongside the stability of the water–oil interface. Furthermore, the surfactant mixture (BrijL23 and Span 80) used in the optimal nanoemulsion might also influence the water droplets' permeability or release. This led to the controlled mass transfer of the encapsulated gallic acid and catechin from the optimal nanoemulsion through the Strat-M membrane, as similarly reported by recent studies^42,44^. In this study, the surfactant mixture (29%) is higher than the oil phase (10%) in the optimal nanoemulsion. This feature was important to delay the rapid mass permeation of the encapsulated polyphenolic/hydrophilic compounds in the water phase (58%).

**Figure 5:**
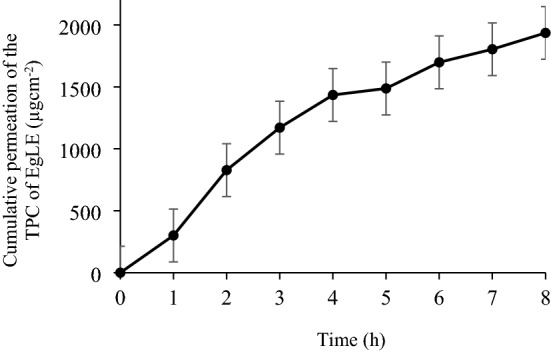
Cumulative amount of TPC of EgLE permeated from the optimal nanoemulsion after 8 h.

Recently, Strat-M membrane is widely used as a synthetic membrane for *in*-*vitro* permeability assay to simulate human skin. The polyethersulfone layer is more resistant to diffusion, whereas the polyolefin layer is more diffusive^45^. On the other hand, the receptor medium contained 50% ethanol because it acts as a permeation enhancer^46^. The graph for the cumulative amount of the TPC of EgLE permeated per unit area versus time was plotted as depicted in Fig. 5. The total amount of the TPC of EgLE permeated across the membrane per unit area after 8 h of study is 1935 ± 45.7 µgcm^−2^, with the steady-state flux (Jss) of 241.9 ± 5.7 µgcm^−2^ h^−1^ and permeation coefficient (Kp) value of 1.15 ± 0.03 cm.h^−1^.

### Kinetic release study

**Figure 6:**
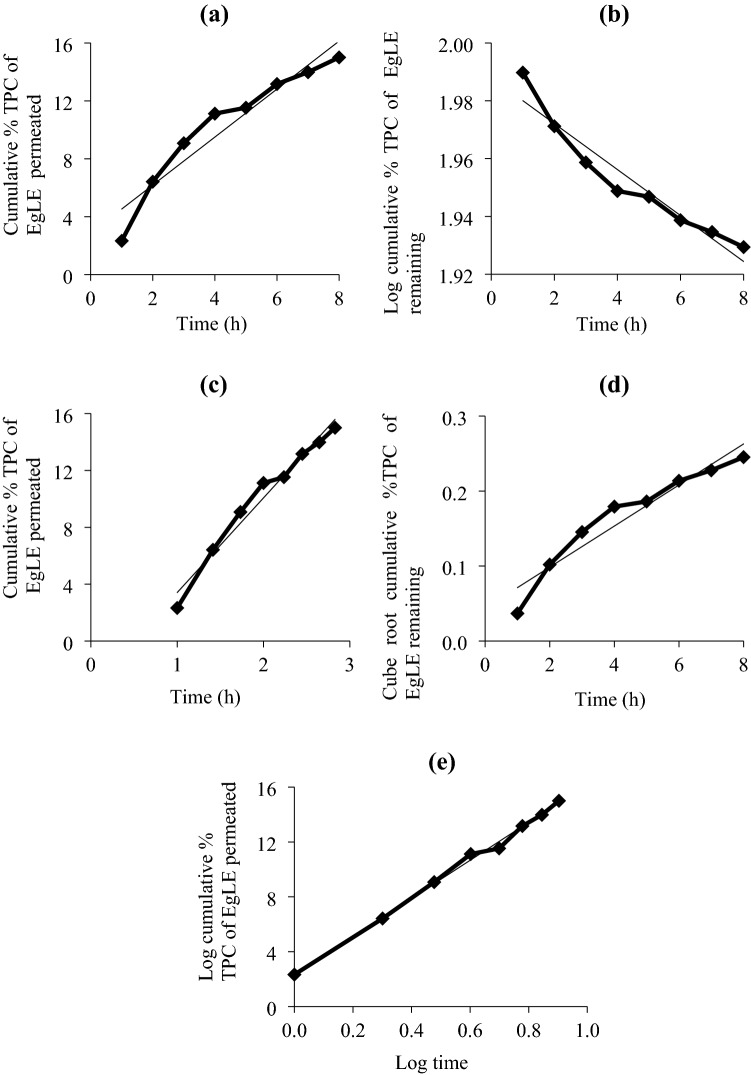
Graph for (**a**) zeroth order, (**b**) first-order, (**c**) Higuchi, (**d**) Hixson–Crowell, and (**e**) Korsmeyer–Peppas for the kinetic release of the TPC of EgLE from the optimal nanoemulsion.

In this study, the kinetic release study was performed to determine a possible release mechanism and best fitted kinetic release models viz. zeroth-order, first-order, Higuchi, Hixson-Crowell, and Korsmeyer-Peppas for the optimal nanoemulsion. The best fitted kinetic release model was chosen based on the coefficient of determination (R^2^) approaching 1. The R^2^ for all the five kinetic release models for the TPC of EgLE from the optimal nanoemulsion is tabulated in Table 1, and Fig. 6 shows the plotted graph for (a) zeroth order, (b) first-order, (c) Higuchi, (d) Hixson-Crowell, and (e) Korsmeyer-Peppas models.

**Table 1:** The R^2^ of all kinetic release models for the TPC of EgLE from the optimal nanoemulsion.

Coefficient of determination (R^2^)	Kinetic release models
0.9144	Zeroth-order
0.9252	First-order
0.9217	Hixson–Crowell
0.9725	Higuchi
0.9961	Korsmeyer–Peppas

In this study, the kinetic release mechanism of the optimal nanoemulsion was best fitted to the Korsmeyer–Peppas model, as seen from the high linearity (R^2^ = 0.9961) (Fig. 6). According to the equation, Korsmeyer–Peppas equation described the type of diffusion by the release exponent, *n* = 13.867, higher than 1. The findings implied that the release of the TPC (gallic acid and catechin) of EgLE from the optimal nanoemulsion exhibited a supercase II transport behavior^14^. The outcome meant that the release of the TPC of EgLE from the optimal nanoemulsion was controlled by the swelling and relaxation of the crosslinked network of the thickener (HPMC), surfactant mixture, and oil phase. The diffusion rate of the TPC of EgLE was also governed by the concentration gradient and polymer relaxation resulting from water uptake and the osmotic pressure occurring during the swelling process^45^.

### Characterization of the AQP-3 protein

The AQP-3 Protein (EC 2.7.11.11) has 292 amino acid residues, and the BRENDA analysis revealed it to be a true protein. The Protparam analysis of the AQP-3 protein primary structure seen in Table 2 showed the amino acid sequence of the AQP-3 protein to comprise 4461 atoms with a corresponding molecular formula and molecular weight of C_1459_H_2233_N_373_O_383_S_13_ and 31543.83 Daltons, respectively. Notably, a computed theoretical pI value below 7 indicates acidic characters, whereas the AQP-3 protein has a low theoretical pI (6.74). This indicated the acidic nature of the AQP-3 protein, which correlated with the high number of acidic residues (Asp + Glu = 16).

**Table 2:** Summary of physicochemical properties of AQP-3 protein determined by the ExPASy ProtParam program.

Details	AQP-3 protein
Amino acid residues	292
Molecular weight (Da)	31,543.83
Theoretical pI	6.74
Negatively charged residues	16
Positively charged residues	15
Molecular formula	C_1459_H_2233_N_373_O_383_S_13_
Total number of atoms	4461
Aliphatic index (%)	105.86
Instability index (%)	22.68
GRAVY	0.535

Additionally, the instability index of AQP-3 protein, 22.68, conveyed that the protein is stable *in*-*vitro* and has a high aliphatic index of 105.86. This high index verifies the protein's thermal stability, as stability- and aliphatic indices of < 40 and > 40, respectively, are the collective attributes of a thermally stable protein^47^. The negative values of the grand average of hydropathicity (GRAVY) of AQP-3 protein (0.535). This value signified that the AQP-3 protein is hydrophobic. Notably, GRAVY is used to measure the hydrophobicity and solubility of protein^18^. A negative GRAVY value denotes hydrophilic protein, while a positive value indicates a hydrophobic protein.

**Figure 7:**
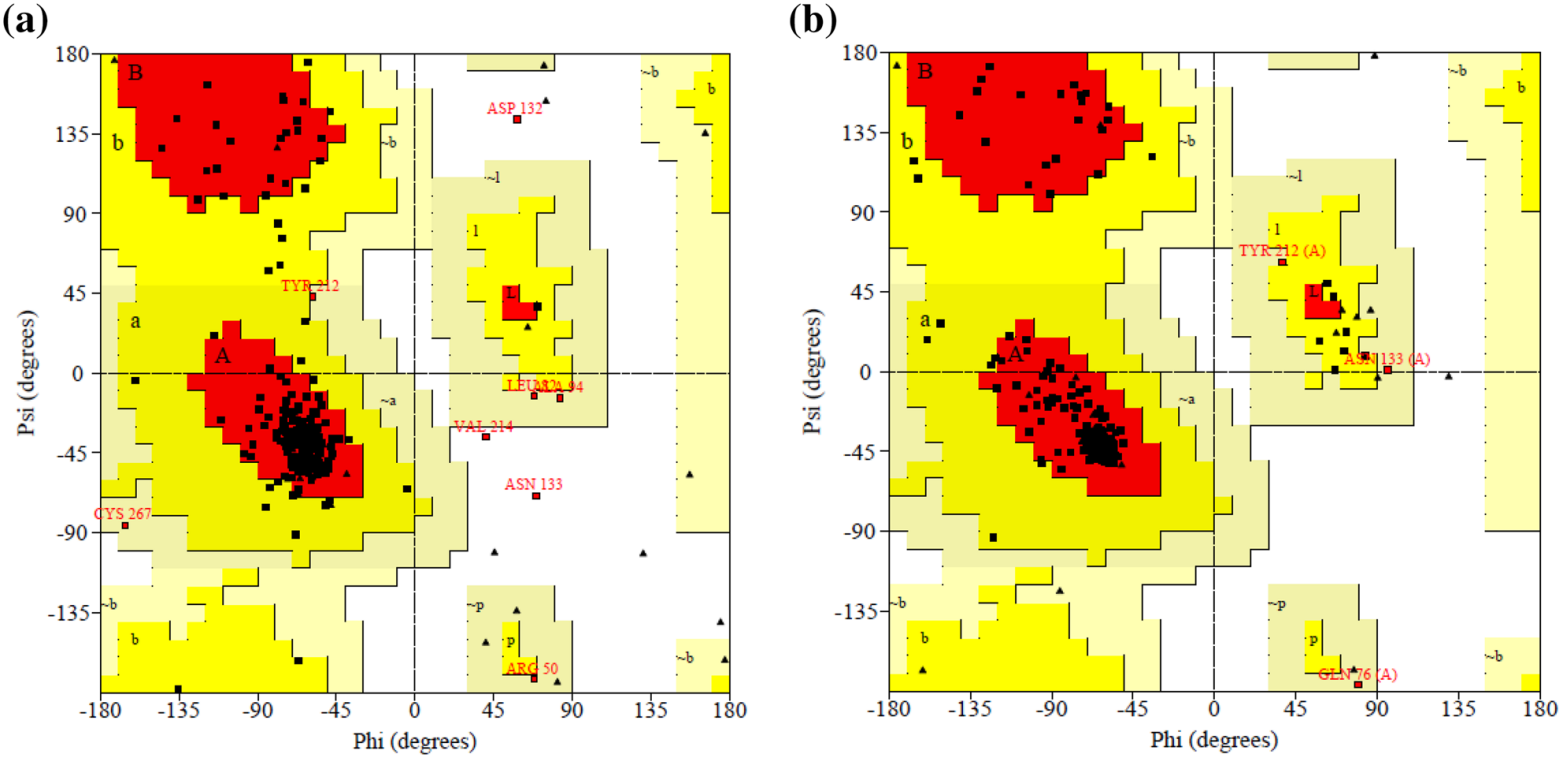
Ramachandran plots of a polypeptide backbone torsion angles psi (w) versus phi (u) of amino acids in the SWISS-MODEL-generated AQP-3 protein homology model generated (**a**) before and (**b**) after energy minimization.

Analyses of the quality of the modeled structure of AQP-3 protein (before and after energy minimization) were performed using PROCHECK, ERRAT, and Verify3D. Meanwhile, the homology model of the AQP-3 protein PROCHECK-generated Ramachandran plots analyzed the polypeptide backbone torsion angles phi (U) and psi (w) of amino acids. The results of the Ramachandran plot (Fig. 7) before and after energy minimization are summarized in Table 3. Residues in the most favorable region were high at 91.2%, followed by 7.3% in the additional allowed region, 1.5% in the generously allowed region, and no residue was found in the disallowed region after energy minimization. Additionally, 100% of the non-glycine and non-proline residues were distributed within the allowable regions. Thus, a good quality protein homology model should have over 90% of residues residing in the most favorable region^47^. Hence, the 3D model of AQP-3 protein was proven stereochemically satisfactory.

**Table 3:** Results of structural validation of AQP-3 protein model with the SAVEs Server (before and after energy minimization).

Validation	Parameter	Scheme	Validation of analysis (%)		
			Before	After	Ranges Score
Procheck	Residues in most favoured regions [A,B,L]	Stereochemical quality	85.4	91.2	> 90
	Residues in additional allowed regions [a,b,l,p]		10.7	7.3	
	Residues in generously allowed regions [~ a, ~ b, ~ l, ~ p]		2.4	1.5	
	Residues in disallowed regions		1.5	0.0	
	Number of non-glycine and non-proline residues		100	100	
ERRAT		Over quality factor	95.73	99.16	> 50
Verify-3D		Amino acid compatibility	81.27	84.46	> 80

**Figure 8:**
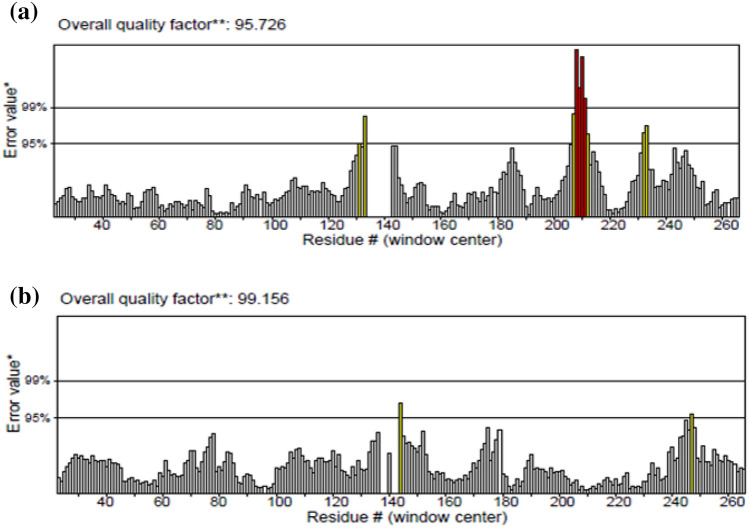
Overall quality of the AQP-3 protein model evaluated by the ERRAT, (**a**) before and (**b**) after energy minimization.

The general quality of the AQP-3 protein 3D model was measured using ERRAT, wherein the ERRAT was 99.16% (Fig. 8). Literature has shown that a good protein model should have an ERRAT score of > 50% (15,48). Meanwhile, the ERRAT histogram illustrates the correct regions as grey, and the incorrect regions are colored black; white bars signify the region with a lower error rate of protein folding. The data revealed that the two lines were drawn at 95% and 99% as the confidence level to reject the regions which exceeded the latter error value. This meant that the energy minimization step improved the structural quality of the 3D model of the AQP-3 protein. The results thus corroborated the excellent quality of the predicted AQP-3 protein structures (> 50%) and therefore are reliable for subsequent structural analysis.

**Figure 9:**
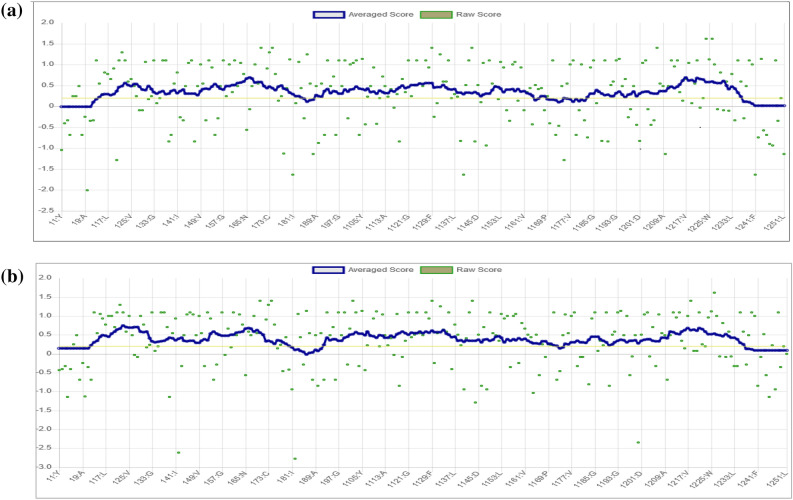
The results for Verify3D for AQP-3 protein model (**a**) before (**b**) after energy minimization.

Consequently, the local environment of the AQP-3 protein was checked using Verify-3D. The program determines the compatibility of an atomic model (3D) with its own amino acids sequence. It is germane to indicate here that a satisfactory protein model has a Verify-3D score of > 80%^48^. Since the Verify-3D scores (Fig. 9) for the AQP-3 protein after energy minimization scored 84.46%, this indicated that the side-chain environments of each residue in the AQP-3 protein model were satisfactory (Table 3).

### Molecular docking

A molecular docking study was attempted to examine the in-silico ability of the ligands (gallic acid and catechin) in the optimal nanoemulsion to bind with the AQP-3 protein to improve skin moisturization and it was done using the AutoDock Vina tool. Here, binding energies, binding interactions, and the AQP-3 protein binding sites to gallic acid and catechin were monitored and compared to gauge the protein-substrates complexes' binding quality. Therefore, the ligands have to bind to AQP-3 protein to increase the expression of the proteins to hydrate the human skin better.

Similarly, a patent (FR2874502A1 dated 26th August 2004) explained the effect of different ligands from pomegranate extract on the expression of AQP-3 protein. It revealed that the pomegranate extract induced the expression of the AQP-3 protein for the moisturization effect, which is an important criterion for cosmetic products. On the other hand, the binding energies of AQP-3 protein with compounds through computational modeling for skin cancer were also investigated^49^. The study found that the two ligands' viz. gallic acid and catechin binding energies were − 5.6 kcal/mol and − 7.4 kcal/mol, respectively (Table 4), thus showing a higher preference of the latter to bind to the AQP-3 protein.

**Table 4:** Summary of docking analysis of AQP-3 protein with gallic acid and catechin from AutoDock Vina tool.

Polyphenolic compounds	Binding energy (kcal/mol)	Number of H-bonds	Crucial interacting amino acids	Distance (Å)
Gallic acid	− 5.6	1	Asn 215	3.04
Catechin	− 7.4	5	Arg 95	3.01
			Ser 78	2.76
			Glu 28	3.06
			Arg 23	2.92
			Gln 24	2.86

**Figure 10:**
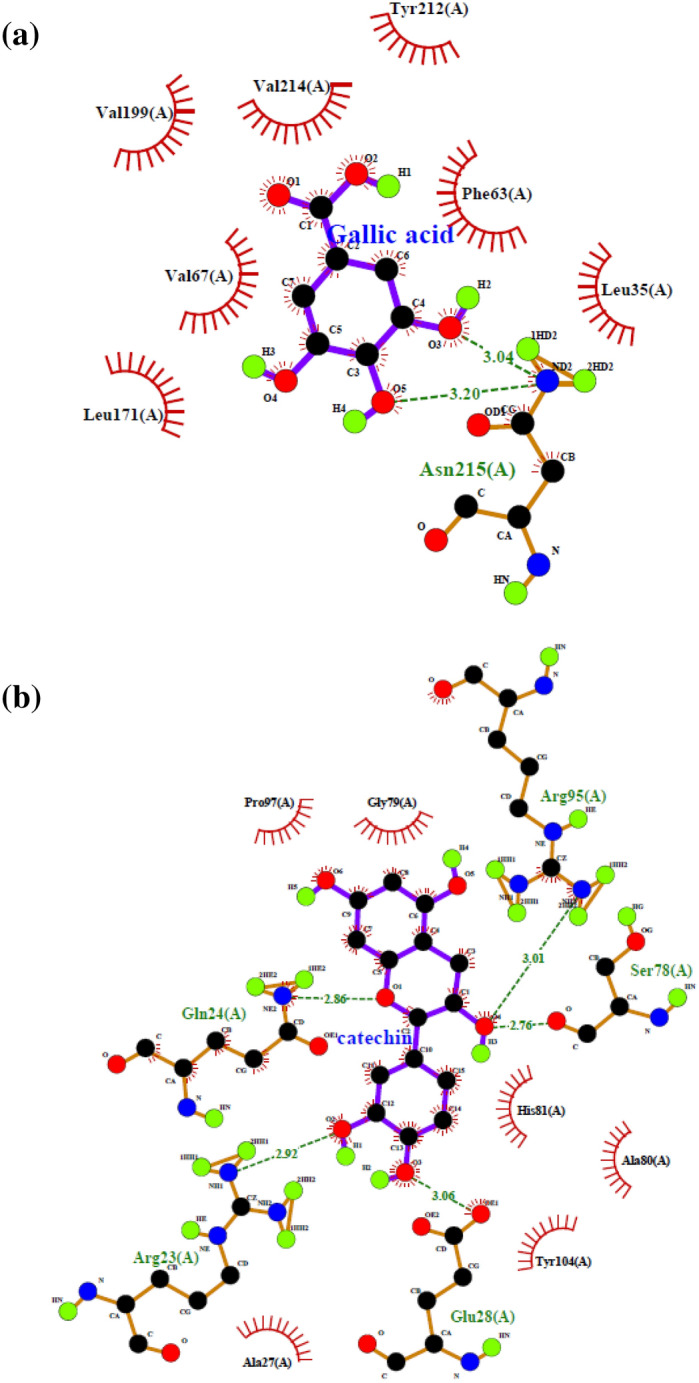
LigPlot for the interactions in the AQP-3-ligand complexes showing hydrogen bond with their equivalent distance and non-ligand residues involved in the hydrophobic interaction (**a**) gallic acid and (**b**) catechin.

It is important to note that the lowest binding energy (kcal/mol) represents a simple estimate of a better or stronger substrate affinity towards the protein for protein-substrate conformation^16^. Also, the protein–ligand complex's specific docking helps predict the ligand's preferred orientation when bound to the protein^15^. In this case, a stronger moisturization effect is expected by the preferable binding of catechin to the AQP-3 protein over gallic acid. The effect was inferred from the higher number of intermolecular interactions between the AQP-3-catechin complex. Figure 10 showed the corresponding LigPlot analysis of the AQP-3-ligand complexes. Gallic acid was bonded to the AQP-3 protein by a single amino acid, Asn 215, via hydrogen and hydrophobic interaction. While more amino acid residues interacted with catechin through five hydrogen bonds via Arg 95, Ser 78, Glu 28, Arg 23, and Gln 24 located nearby the catalytic site of AQP-3 protein.

Based on Table 4, the lowest binding energy for the AQP-3-catechin complex (− 7.4 kcal/mol) indicated a stronger AQP-3-ligand complex interaction compared to gallic acid (− 5.6 kcal/mol). The lower binding energy of the AQP-3-catechin complex corresponded well with the five hydrogen bonds that held the complex together at the distances of 3.01 Å, 2.76 Å, 3.06 Å, 2.92 Å, and 2.86 Å. Conversely, the AQP-3-gallic acid complex's higher binding energy contributed to a single hydrogen bond with a distance of 3.04 Å to Asn 215. It is pertinent to indicate here that < 3.5 Å is the cut-off distance for an intermolecular hydrogen bond. Hence, a longer bond distance (> 3.5 Å) conveys a lower binding affinity of a protein towards a ligand and is less likely to bind the ligand.

### Molecular dynamics simulation

MD simulation is crucial in silico investigation to demonstrate and analyze the behavior, structural flexibility, and stability of the protein when bonded to different ligands^50^. In this study, the structural changes, stability, and flexibility of AQP-3 protein with the ligands (gallic acid and catechin) were monitored by comparing the values of RMSD, RMSF, Rg, and hydrogen bonds distance. The following results were the mean value of triplicated analyses of the AQP-3-ligands complexes simulated for 100 ns.

#### Root mean square deviation

**Figure 11:**
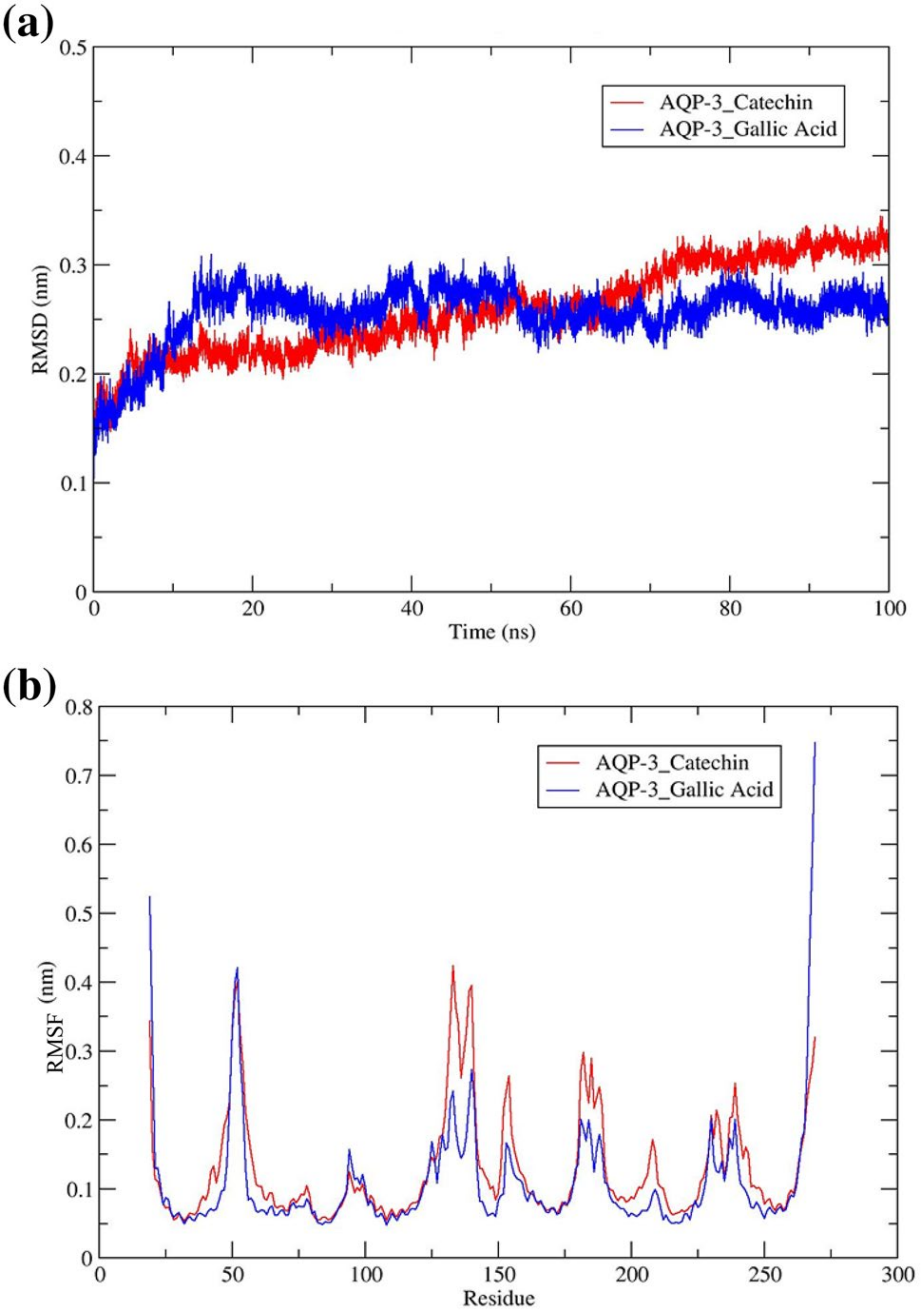
Average (**a**) RMSD and (**b**) RMSF for AQP-3-ligands (gallic acid and catechin).

It is important to indicate here that a low RMSD value (< 0.3 nm) conveys a strong binding affinity and the formation of a stable complex^51^. The analysis of RMSD of the AQP-3 protein backbone was calculated to describe the conformational changes of the two different ligands, gallic acid and catechin. As can be seen, the low RMSD values (RMSD ~ 0.1–0.35 nm) for the AQP-3-catechin and AQP-3-gallic acid complexes indicated the strong binding between the two complexes, with the former being a stronger one. The outcome was suggestive of better skin hydration through catechin in the optimal nanoemulsion. The RMSD value of the AQP-3-gallic acid complex reached equilibration considerably earlier after 50 ns at ~ 0.25 nm than the AQP-3-catechin complex, which fluctuated for 75 ns before achieving equilibrium around 0.30 nm during the production simulation (Fig. 11a).

Based on the MD simulation results, although AQP-3 protein binds preferably to catechin over gallic acid, as shown in the molecular docking studies (Table 4), it took a while to form the stable AQP-3-catechin complex. It was plausible that a longer duration was needed for the five hydrogen bonds to bind the two moieties than only a single hydrogen bond in the AQP-3-gallic acid complex. The results conveyed that the moisturizing ability of the optimal nanoemulsion was contributed by the rapid action of catechin to bind to the AQP-3 protein. Although gallic acid eventually bonded to the AQP-3 protein, the rate was slower than that of catechin. Similarly, *α*-glucosidase bonded more favorably to catechin than gallic acid. This was reported by Choudhary et al., where the RMSD observed was among the more prevalent MD trajectories for the catechin-*α*-glucosidase and the gallic acid-*α*-glucosidase, corresponding to 0.2 and 0.3 nm, respectively. They confirmed that catechin inhibited *α*-glucosidase activity more efficiently than gallic acid^52^.

#### Root mean square fluctuation

RMSF analysis imparts information on residue-specific flexibility as the parameter calculates the individual residue flexibility or the extent of any particular residue moves (fluctuates) during an MD simulation. The prediction was made by measuring the amount of movement along a principal axis. It is important to indicate here that the highest value of RMSF represents a higher degree of movement, whereas the lowest RMSF value implies a more stable structure from limited structural fluctuation during the MD simulation^53,54^. In this study, the average RMSF plot (Fig. 11b) showed moderately high structural stability levels for the complexes in the range of 0.05–0.45 nm.

Notably, the RMSF value of the AQP-3-gallic acid complex was slightly lower than that of the AQP-3-catechin complex, thus indicating that the gallic acid bonded more tightly to AQP-3 protein at residue positions, 128, 135, 153, 180, 215, and 230. The data demonstrated that AQP-3 protein is bonded slightly less flexible with catechin, which is in good correlation with the RMSD values in the previous subsection 3.10.1, exhibiting the fast equilibrating AQP-3-gallic acid complex (50 ns) over the AQP-3-catechin complex (75 ns). This could be explainable that the gallic acid is much smaller in structural size compared to catechin. This would limit the structural movement and flexibility of gallic acid upon binding with the AQP-3 protein. Albeit the overall RMSF values indicated that both the complexes exhibited low structural movements and flexibilities during simulation trajectories, it is found that the AQP-3-catechin complex formed more stable binding and increased the expression of AQP-3 protein for better skin hydration. Similarly, Anuar et al. concluded earlier that the mutated LipKV1-tributyrin exhibited greater stability at pH 8.0 because it recorded a lower RMSF value than the LipKV1-tributyrin complex^15,16^.

#### Radius of gyration

**Figure 12:**
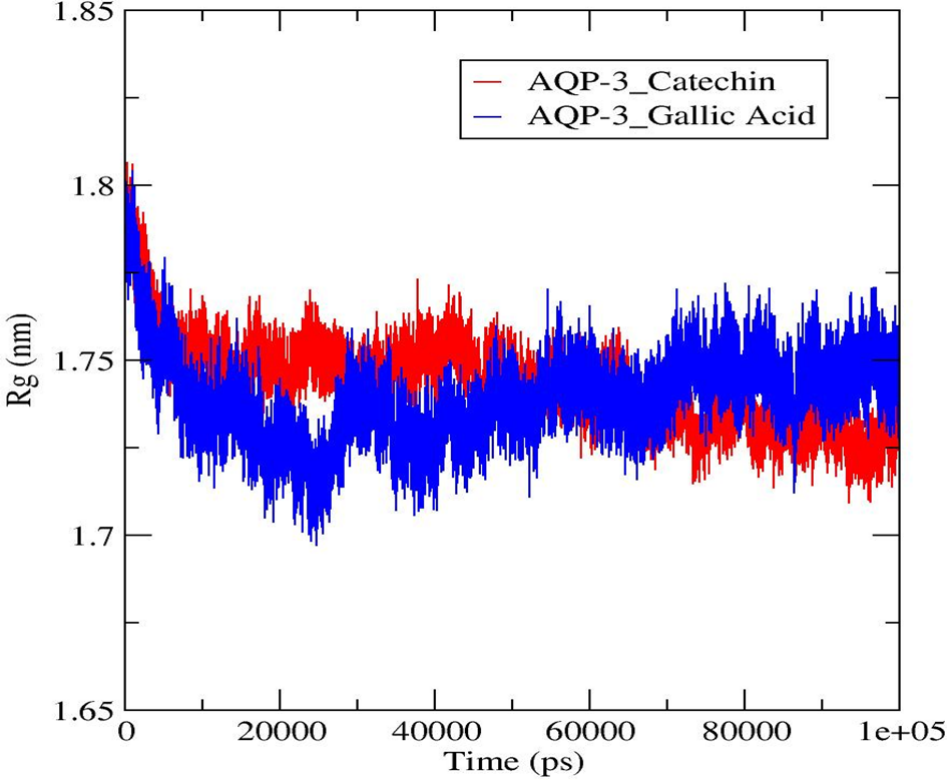
Average Rg value for the AQP-3-gallic acid and -catechin complexes.

In this investigation, the average Rg value of the AQP-3 complexes fluctuated in the range between 1.70 nm–1.80 nm (Fig. 12). Remarkably, AQP-3-catechin decreased from 30,000 ps onwards before achieving equilibration with small fluctuations at ~ 1.755 nm. Contrariwise, the AQP-3-gallic acid complex fluctuated before reaching equilibrium at 1.75 nm from 70,000 ps onwards. The catechin and gallic acid were loosely packed with the AQP-3 amino acid residues based on the Rg values. These AQP-3-ligands are less compact, hence can move more freely. According to the literature, the highest Rg values indicate a looser packing of amino acids with ligands and vice versa. Likewise, Rg values for all the DehH2-ligand studied fluctuated between 1.75–1.85 nm. These values revealed less compact conformation of the DehH2-ligand complex, which conformed to the poor binding to the enzyme's catalytic residues^17,55^.

#### Hydrogen bonds analysis

**Figure 13:**
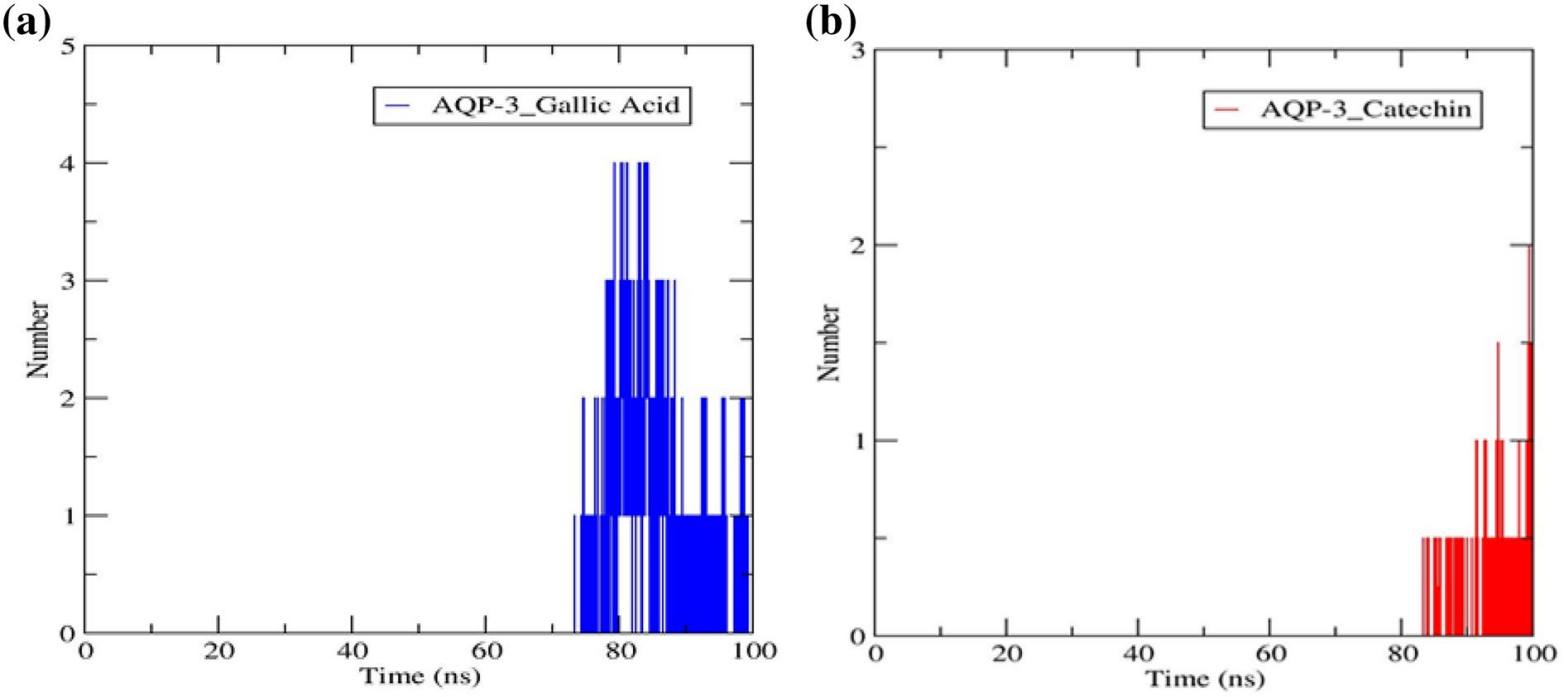
Intermolecular hydrogen bonds along 100 ns simulation time in the AQP-3 protein when chemically bonded to (**a**) gallic acid (**b**) catechin.

Hydrogen bonds and their relative strength in a water environment are vital to enable protein–ligand binding. This is particularly true when the mechanism of action involves hydrolysis, where water plays an important role in the compound's breakdown. Hydrogen bonds are formed when an electronegative atom of a hydrogen-bond acceptor binds to a hydrogen atom directly bonded to a hydrogen-bond donor^56^. Figure 13 depicted the number of hydrogen bonds in the AQP-3-gallic acid and –catechin complexes. Catechin appeared to bind strongly to AQP-3 protein compared to gallic acid, despite the formed four hydrogen bonds at 70 ns in the latter, before stabilizing to a two-hydrogen bonded AQP-3-catechin complex from 88 ns onwards. The study proved that the AQP-3-catechin complex was stabilized much later, equilibrating at a two-intermolecular hydrogen-bonded complex from 88 ns onwards.

The AQP-3-gallic acid output seen in this study exhibited a higher number of intermolecular hydrogen bonds than the corresponding docking study in subsection 3.9. On the other hand, there were only two hydrogen bonds in the AQP-3-catechin complex instead of five, as predicted by molecular docking. The findings are somewhat justifiable that water molecules were included in the system during MD simulation. Hence, the produced output is a close resemblance to the actual protein hydrolysis system. Therefore, AQP-3-catechin exhibited stronger hydrogen bonding in comparison with AQP-3-gallic acid. The results proved that catechin was the better polyphenolic compound to hydrate the skin. The earlier study also observed the same outcome wherein the number of hydrogens changed throughout the simulation, but the changes were more profound^17^.

#### MM-PBSA binding free energy

Based on the previous studies, data derived from the average binding energy (kcal/mol) calculations can offer a better insight into the protein–ligand complexes' interactions. Consequently, the binding strengths between the AQP-3 protein and the tested ligands (gallic acid and catechin) were assessed through binding free energy calculation via MM-PBSA based on MD trajectories of van der Waals, electrostatic, polar solvation, and nonpolar solvation energies. MM-PBSA offered more reliable and accurate predictions on AQP-3-gallic acid and –catechin complexes than molecular docking. This is because of the molecular docking system's rigidity in the absence of water molecules that limit the protein–ligand binding's flexibility to certain types of motions. Moreover, the energy derived from MD trajectories is more responsive and flexible when the protein–ligand complex is interchange effectively throughout 100 ns simulation^15,17^.

As tabulated in Table 5, AQP-3-catechin complex exhibited the most favorable binding energy, − 57.514 kcal/mol compared to AQP-3-gallic acid (− 8.553 kcal/mol). The data corresponded with the AQP-3-gallic acid complex's highest van der Waals energy (− 0.063 kcal/mol) compared to AQP-3-catechin (− 63.335 kcal/mol). The highest electrostatic energy of AQP-3-gallic acid (− 0.108 kcal/mol) indicated that this complex is less positively charged compared to AQP-3-catechin, which showed the lowest electrostatic energy (− 1.985 kcal/mol)^16^. As can be seen, the MM-PBSA calculation validated MD simulation data where catechin interacted spontaneously with the AQP-3 protein, as seen by the negative binding value of Gibbs free energy. The more negative binding energy is an indication that the reactions are spontaneous. Hence, the more negative the reactants' electrostatic energy, they tend to undergo reactions without an external energy source^15^. In this milieu, the protein–ligand complex is converted quicker into the product at a higher reaction rate. In conclusion, AQP-3 protein prefers to bind with catechin to increase the expression of AQP-3 protein for skin hydration.

**Table 5:** Binding free energies from MM-PBSA in kcal/mol for AQP-3-ligands complexes.

Energy components (kcal/mol)	AQP-3-gallic acid	AQP-3-catechin
∆E_vdW_	− 0.063	− 63.335
∆E_ele_	− 0.108	− 1.985
∆G_polar_	− 8.631	− 6.348
∆G_nonpolar_	0.249	− 8.543
∆G_binding_	− 8.553	− 57.514

## Conclusion

The optimal nanoemulsion was not influenced by the coalescence, but it was under significant influence of the Ostwald ripening effect during the 90 days of storage stability monitoring at 25 °C. On the other hand, the in-vitro permeability revealed a controlled and sustained release of the TPC of EgLE from the optimal nanoemulsion. The cumulative amount of the TPC of EgLE permeated across the membrane was 1935 ± 45.7 µgcm^−2^ after 8 h of study. Whilst, the steady-state flux (Jss) and permeation coefficient (Kp) values were 241.9 ± 5.7 µgcm^−2^ h^−1^ and 1.15 ± 0.03 cm.h^−1^, respectively. The study identified Korsmeyer–Peppas model as the best fitted kinetic release model for the optimal nanoemulsion with an R^2^ value of 0.9961, which pointed towards a super case II transport mechanism. The *in*-*silico* molecular docking, MD simulations, and MM-PBSA results verified that the AQP-3-catechin complex was the preferred complex, followed by the AQP-3-gallic acid complex. The conclusion was based on their corresponding lowest binding energy of − 57.514 kcal/mol compared to − 8.553 kcal/mol in the latter. Thus, catechin has a more promising moisturization capacity based on the higher binding affinity to form hydrogen bonds with the AQP-3 protein, as predicted by this in-silico study. Nevertheless, in-vivo transepidermal water loss (TEWL) and moisture tests should be conducted to support further or validate this in-silico molecular modeling study. In a nutshell, the optimal nanoemulsion developed in this study is predicted to be a promising moisturizer for topical application.
